# Effect of High-Flux Dialysis on Circulating FGF-23 Levels in End-Stage Renal Disease Patients: Results from a Randomized Trial

**DOI:** 10.1371/journal.pone.0128079

**Published:** 2015-05-29

**Authors:** Andreas Schneider, Markus P. Schneider, Detlef H. Krieter, Bernd Genser, Hubert Scharnagl, Tatjana Stojakovic, Christoph Wanner, Christiane Drechsler

**Affiliations:** 1 Department of Medicine, Division of Nephrology, University Hospital, Würzburg, Germany; 2 Department of Nephrology and Hypertension, University Hospital, Erlangen-Nürnberg, Germany; 3 Mannheim Institute of Public Health, Social and Preventive Medicine, Medical Faculty, Mannheim, Germany; 4 BGStats Consulting, Vienna, Austria; 5 Medical University of Graz, Clinical Institute of Medical and Chemical Laboratory Diagnostics, Graz, Austria; L' Istituto di Biomedicina ed Immunologia Molecolare, Consiglio Nazionale delle Ricerche, ITALY

## Abstract

**Background:**

In patients undergoing maintenance hemodialysis (HD), increased levels of circulating fibroblast growth factor-23 (FGF-23) are independently associated with cardiovascular events and mortality. Interventional strategies aiming to reduce levels of FGF-23 in HD patients are of particular interest. The purpose of the current study was to compare the impact of high-flux versus low-flux HD on circulating FGF-23 levels.

**Methods:**

We conducted a post-hoc analysis of the MINOXIS study, including 127 dialysis patients randomized to low-flux (n = 62) and high-flux (n = 65) HD for 52 weeks. Patients with valid measures for FGF-23 investigated baseline and after 52 weeks were included.

**Results:**

Compared to baseline, a significant increase in FGF-23 levels after one year of low-flux HD was observed (Delta plasma FGF-23: +4026 RU/ml; p < 0.001). In contrast, FGF-23 levels remained stable in the high flux group (Delta plasma FGF-23: +373 RU/ml, p = 0.70). The adjusted difference of the absolute change in FGF-23 levels between the two treatment groups was statistically significant (p < 0.01).

**Conclusions:**

Over a period of 12 months, high-flux HD was associated with stable FGF-23 levels, whereas the low-flux HD group showed an increase of FGF-23. However, the implications of the different FGF 23 time-trends in patients on high flux dialysis, as compared to the control group, remain to be explored in specifically designed clinical trials.

**Trial Registration:**

German Clinical Trials Register (DRKS) DRKS00007612.

## Introduction

Despite efforts to improve outcomes in patients with end-stage renal disease (ESRD), mortality remains excessively high [1]. Cardiac and vascular events are the predominant causes of death [1, 2]. Recently, non-traditional risk factors and specific biomarkers were identified that distinguish ESRD patients at high risk for CVD and mortality.

Fibroblast-growth-factor-23 (FGF-23) is a 251 amino-acid peptide synthesized by osteocytes and osteoblasts, and is involved in the regulation of phosphate homeostasis [3, 4]. In chronic kidney disease (CKD), FGF-23 increases early in the course of the deterioration of kidney function, and has been proposed to be a physiological response that protects the organism from the adverse effects of phosphate retention by facilitating phosphate excretion [5]. On the other hand, elevated FGF-23 levels were found to be independently associated with mortality in ESRD patients as well as in patients with advanced CKD [6, 7]. Thus, in view of the high cardiovascular risk in CKD and ESRD, strategies that are able to reduce FGF-23 levels are of particular interest. During the past two decades, more permeable high-flux dialysis membranes, which are able to eliminate low-molecular weight toxins up to 50.000 Dalton [8] have been developed. This is of clinical interest as FGF-23 is a free circulating peptide in the blood with a molecular weight of 32.000 Dalton [4, 9] which might be removed by high-flux hemodialysis (HD).

In the MINOXIS study, maintenance HD patients were randomized to 52 weeks treatment with either low-flux or high-flux HD with the primary endpoint being parameters of anemia [10]. In the present post-hoc analysis of the MINOXIS data, effects of high-flux versus low-flux HD on FGF-23 serum levels were compared.

## Methods

The study was performed in accordance with the ethical principles of the declaration of Helsinki. Written informed consent was obtained from each participant before entry into the study. The study was approved by the Freiburger Ethik-Kommission International (FEKI Nr.: 04/1952) and was conducted according to “good clinical practice” (GCP) guidelines. The study is registered in the „German Clinical Trials Register (DRKS)”(trial registry number DRKS00007612).

### Study design

**Fig 1:**
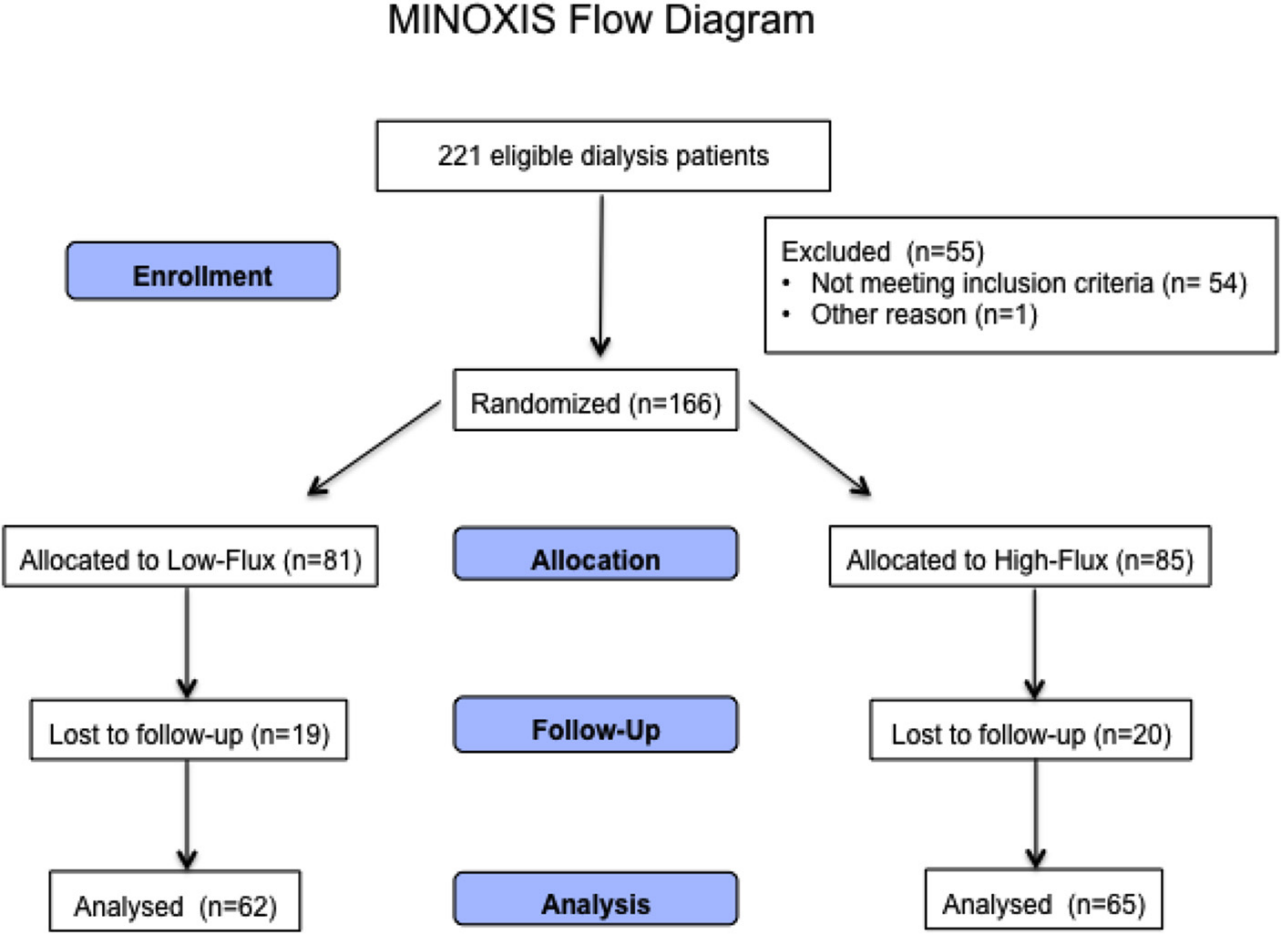
Flow chart of the MINOXIS study.

The MINOXIS study design, main outcome findings, and baseline data have been described previously [10]. In summary, MINOXIS was a prospective randomized controlled trial recruiting 166 patients with ESRD on maintenance HD (Fig 1). Included patients had to be ≥ 18 years of age and receiving treatment for anemia with the erythropoietin stimulating agent (ESA) darbepoetin-alfa for at least 6 months. Incident patients undergoing HD for at least 1 or at most 3 months (recently diagnosed) had to be treated by low-flux dialysers. Prevalent patients undergoing HD for more than 3 months had to be treated with low-flux dialysers for at least 3 months before inclusion in the study. Exclusion criteria were serious comorbidities with a life expectancy of less than 2 years, single-needle HD, use of temporary or permanent catheters, planned kidney transplantation or pregnancy. Patients initially underwent a run-in period of 20 weeks, in which they received HD treatment with low-flux dialysers. Upon completion of week 20, patients were randomly assigned to one of the two treatment groups (low-flux or high-flux dialysis, 1:1 ratio). To be eligible for randomization, patients had to have a high-sensitive C-reactive protein (hsCRP) level of < 50 mg/l and a hemoglobin concentration within the target range of 10.0–13.0 g/dl. After randomization, patients were followed-up for 52 weeks (main study period). The study had a total duration of 2 years and 5 months. As regards the main outcome parameters, high flux dialysis had no superior effects on hemoglobin levels or markers of inflammation, oxidative stress, and nutritional status [10].

### Study dialyzers and HD treatment

In the main study, synthetic polysulfone dialysers (Fresenius Medical Care Deutschland GmbH, Bad Homburg) were used exclusively. For patients on low-flux dialysis, surface areas between 1.3 and 2.4 m^2^ were used (Fresenius Hemoflow F6HPS to F10HPS, steam sterile). High-flux dialysis was administrated with surface areas between 1.0 and 2.4 m^2^ (Fresenius FX50 to FX100, steam sterile). All patients had received HD before inclusion in the study essentially via an arterio-venous fistula (low-flux 91.9%, high-flux 89.2%). HD treatments were performed three times per week, each with a duration of at least 3 hours.

### Data collection

Information on demographic characteristics, such as age, sex and, smoking status, were obtained. Comorbidities including coronary heart disease (CHD), myocardial infarction as well as duration of diabetes and HD treatment were reported by the patients`nephrologists. Blood samples for routine analyses (e.g. albumin, C-reactive protein, phosphate, calcium, haemoglobin etc.) were measured at local laboratories.

At the time of randomization (Month 0) and at completion of the study (Month +12), additional blood samples were drawn for FGF-23 and bone-specific alkaline phophatase (BAP). These blood samples were frozen at -20°C. Serum FGF-23 (C-term) was measured by a second generation two-site enzyme-linked immunosorbent assay (ELISA, Immuntopics International, Can Clemente, CA, US). Serum BAP was measured using a sandwich ELISA (Immundiagnostic GmbH, Bensheim, Germany). The coefficient of variation (CV) were as follows: (i) FGF-23: intra-assay CV 3.4%, inter-assay CV 5.1%; (ii) BAP: intra-assay CV 3.5%, inter-assay CV 5.4%. All blood samples in our study were drawn pre-dialysis.

### Statistical analysis

We calculated descriptive statistics (means and standard deviation, or median and interquartile range) for continuous variables, and frequency tables and percentages for categorical variables. The primary endpoint of this analysis was the difference between the high-flux and low-flux group in the change of FGF-23 concentrations between baseline (month 0) and end of follow-up (month 12) measurements. As secondary endpoints we analyzed the concentration of specific biomarkers of mineral and bone metabolism, namely PTH, BAP, calcium, phosphate and 25-hydroxyvitamin D levels. Similar to calculations for the primary endpoint, the intra-individual changes of PTH, BAP, calcium, phosphate and 25-hydroxyvitamin D concentrations were determined and compared between the two treatment groups. Distributions of absolute differences for all outcome variables were approximately normal distributed, thus all statistical analyses for absolute differences were conducted on the original scale. We used unpaired t-tests to compare the absolute change between the two groups. For multivariate analysis we fitted two different regression models for the absolute difference: i) model A aimed to adjust for regression to the mean by including the baseline measurement as covariate and ii) model B additionally adjusting for a set of potential confounding variables (namely age, sex, C-reactive protein, albumin, calcium, phosphate, 25-hydroxyvitamin D and PTH). For both models we calculated the predicted marginal means of the absolute change evaluated at the mean of the covariates. We considered the full multivariate model B as the core model to test the hypothesis whether there was a difference between the two groups. In addition, we modelled the linear change between baseline and 12 months by a multivariate mixed effect models adjusted for the same covariates as in model B. All statistical analyses were conducted using the statistical software package STATA (StataCorp. 2011. Stata Statistical Software: Release 13. College Station, TX: StataCorp LP).

## Results

### Patient characteristics

Altogether, 166 patients were included into the MINOXIS study, of which 127 patients had valid measures for all bone markers investigated at both evaluation time points (i.e., baseline and 12 months). Table 1 shows the patients baseline characteristics, which were not different between the low-flux and the high-flux groups.

**Table 1:** Baseline characteristics of the study population.

	Low-FluxHemodialysis Group (n = 62)	High-FluxHemodialysis Group (n = 65)	P-value
Demographic variables			
age	66.0 (9.9)	65.5 (12.5)	0.80
age ≥ 70 years (%)	43.5	41.5	0.68
men (%)	47%	63%	0.07
mean height (cm)	167 (8.5)	170 (10.0)	0.09
mean weight (kg)	77 (17.8)	79 (18.2)	0.62
mean systolic BP (mmHg)	137 (19.2)	134 (22.1)	0.40
arterio-venous fistula (%)	92	89	0.60
renal residual function > 300 ml/d	41	38	0.90
Type of primary kidney disease (%)			0.30
diabetic nephropathy	25.8	36.9	
glomerulonephritis	22.6	15.3	
interstitial nephritis	8.1	7.7	
nephrosclerosis	11.3	13.9	
polycystic kidney disease			
Comorbid conditions (%)			
diabetes mellitus	40.3	49.2	0.28
arterial hypertension	77.4	86.2	0.43
CHD	33.9	35.4	0.90
stroke	8.1	10.8	0.60
MI	9.7	10.8	0.58
cardiac arrhythmia	17.7	10.8	0.22
CHF	22.6	20.0	0.59
Mean laboratory values			
hemoglobin (g/dl)	10.4 (2.4)	10.8 (2.2)	0.34
phosphate (mmol/L)	2.1 (0.8)	2.3 (1.4)	0.32
albumin (g/dl)	3.7 (0.6)	3.7 (0.6)	0.71
hs-CRP (mg/L) ^a^	2.9 (7.2)	4.0 (6.7)	0.19
urea predialytic (mg/dl)	95.4 (56.9)	108.3 (52.9)	0.21
urea postdialytic (mg/dl)	28.2 (19.5)	34.9 (22.7)	0.13
creatinine predialytic (mmol/L)	260 (398)	196 (364)	0.38
Mean hemodialysis variables			
duration of hemodialysis (hr)	4.1 (0.5)	4.1 (0.6)	0.71
rate of blood flow (ml/min)	291 (42)	290 (34)	0.96
rate of dialysate flow (ml/min)	497 (26)	500 (0)	0.30
UF volume (ml)	2304 (1224)	2607 (1187)	0.18
Concomitant medication (%)			
antihypertensives	83.6	90.8	0.23
statins	41.0	30.8	0.11
ASS	37.7	32.3	0.53
clopidogrel	6.6	10.8	0.40
warfarin	4.9	10.8	0.23
vitamin D	67.7	56.9	0.21

Values presented with numbers in parentheses are mean (SD). CHD, coronary heart disease; MI, myocardial infarction; CHF, congestive heart failure; hs-CRP, high-sensitivity C-reactive protein; UF, ultrafiltration; HMG-CoA, hydroxymethyl glutaryl coenzyme A; ASS, acetylsalicylic acid.

^a^ Median (interquartile range).

### Primary endpoint; FGF-23

**Fig 2:**
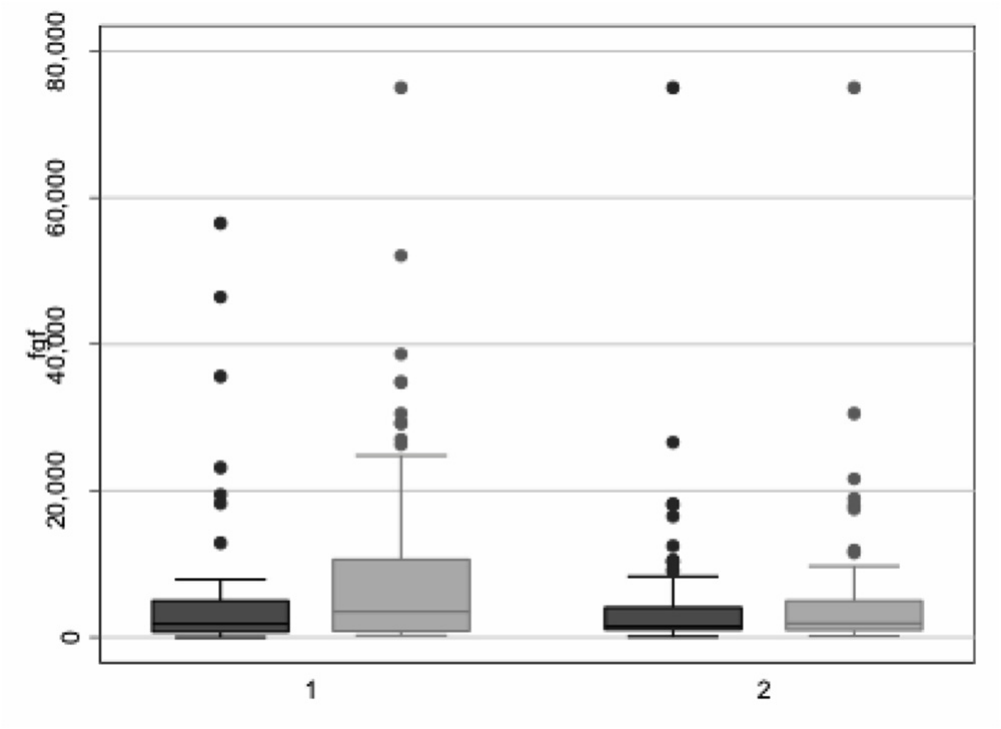
Grouped boxplots visualizing the distributions of FGF-23 before and after the intervention stratified by randomization group. Black box: FGF-23 before the intervention, Grey box: FGF-23 after the intervention. 1: low-flux group, 2: high-flux group.

**Fig 3:**
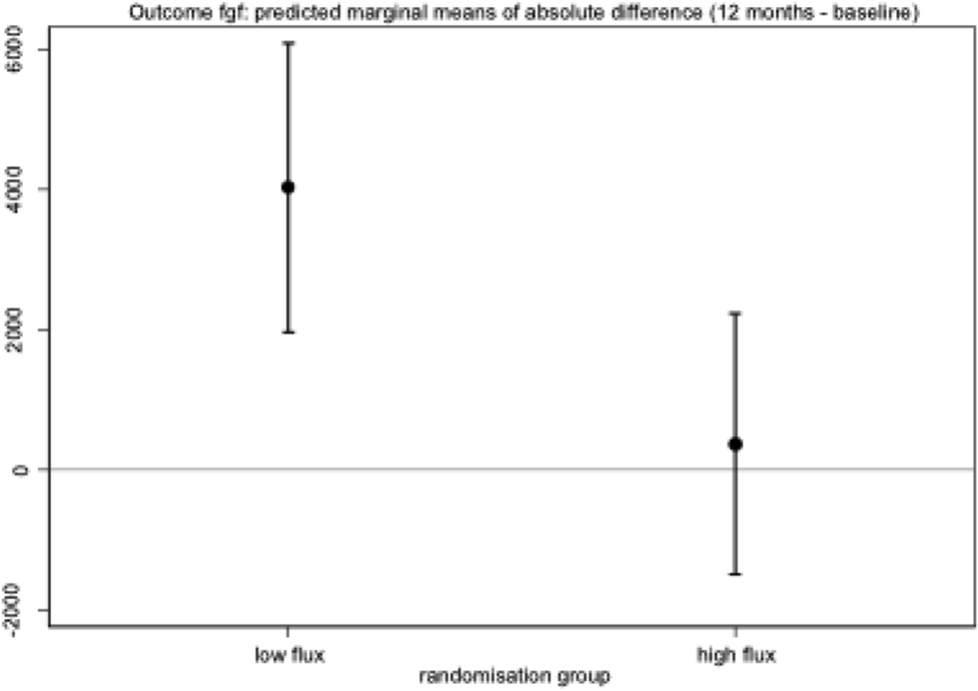
FGF-23; predicted marginal means of the absolute difference (12 months—baseline).

Patients in the low-flux dialysis group had a mean FGF-23 value of 5493 ± 10499 RU/ml at baseline and 9866 ± 14452 RU/ml at the end of the study (p < 0.001, Table 2, S1 Fig). In the high-flux group, mean FGF-23 was 4912 ± 10252 RU/ml at baseline and 5437 ± 10559 RU/ml at the end of the study (p = 0.70, Table 2, S2 Fig). Thus, there was an increase of FGF-23 in the low-flux group but not in the high-flux group during the course of the study. The adjusted difference in the absolute change from baseline between the two groups was 4537 RU/ml (95% CI: 1534 to 7541, p < 0.01) (Figs 2 and 3; Table 2).

**Table 2:** Outcome.

	Low-Flux(Baseline)	Low-Flux(52 weeks)	p-Value	High-Flux (Baseline)	High-Flux(52 weeks)	p-Value	Δ change LF vs HF (adjusted)	p-Value
FGF-23 (RU/ml)	5493 ± 10499	9866 ± 14452	**p< 0.001**	4912 ± 10252	5437 ± 10559	p = 0.70	4537	**P <0.01**
PTH (pmol/l)	152 ± 235	233 ± 354	**p < 0.01**	99 ± 145	147 ± 193	p = 0.08	31	p = 0.74
25(OH)D (ng/ml)	20.4 ± 10.8	22.9 ± 12.3	**p < 0.01**	20.0 ± 20.4	21.3 ± 11.8	p = 0.30	1.8	p = 0.14
Calcium (mmol/l)	2.32 ± 0.18	2.28 ± 0.18	p = 0.93	2.26 ± 0.19	2.22 ± 0.17	**p = 0.01**	0.05	p = 0.13
Phosphate (mmol/l)	2.06 ± 0.78	1.94 ± 1.04	**p < 0.001**	2.28 ± 1.41	1.88 ± 0.73	**p < 0.001**	0.09	p = 0.34
BAP (μg/l)	19.04 ± 20.5	22.6 ± 20.9	p = 0.68	19.9 ± 14.3	24.4 ± 22.4	**p < 0.01**	5.9	P = 0.07

### Secondary endpoints; specific markers of mineral and bone metabolism

There was a significant increase in PTH in the low-flux group during the course of the study, which was not found in the high-flux group (Table 2). However, the changes in PTH were not different between the two groups (p = 0.74). Regarding 25(OH)D levels, there was no difference in the change between the two groups (p = 0.14). We found a significant decrease in serum calcium levels only in the high-flux group (p = 0.01), but again, there was no difference in the change of calcium levels between the two groups (p = 0.13). Serum phosphate levels decreased in both groups during the course of the study. However, there was no difference in the change of phosphate levels between the two groups (p = 0.34). In patients who underwent high-flux dialysis, BAP significantly increased during the study. However, the difference in absolute change between the two groups was not significant (p = 0.07).

### Linear regression models and multivariate mixed effect model

The results of the linear regression models are shown in Table 3. Model A, adjusting for baseline values, demonstrated a significant difference between the low-flux and the high-flux HD groups in the change of FGF-23 levels. There was no significant difference for any of the other parameters examined. Model B, additionally adjusting for age, sex, CPR, albumin, calcium, phosphate, 25-hydroxyvitamin D and PTH, was also significant for the change of FGF-23 levels, but not for any of the other parameters examined. Finally, we also applied a multivariate mixed effects model. Using this approach, a significant interaction between treatment group and measurement time-point was demonstrated for FGF-23 levels (Group: p = 0.78, Measurement: p = 0.22, Group x Measurement: p = 0.05).

**Table 3:** Outcome.

	Low-Flux	High-Flux	Δchange	95% CI	p-value
**FGF-23 (RU/ml)**					
Model A	4392	506	3886	(1035–6737)	**p < 0.01**
Model B	4725	188	4537	(1534–7541)	**p < 0.01**
**PTH (pmol/l)**					
Model A	101	40	61	(25–148)	p = 0.16
Model B	86	54	31	(54–117)	p = 0.74
**25(OH)D (ng/ml)**					
Model A	2.5	1.2	1.3	(1.0–3.6)	p = 0.26
Model B	2.7	0.9	1.8	(0.62–4.2)	p = 0.14
**Calcium (mmol/l)**					
Model A	0.007	0.06	0.05	(0.01–0.11)	p = 0.10
Model B	0.009	0.06	0.05	(0.01–0.11)	p = 0.13
**Phosphate (mmol/l)**					
Model A	0.34	0.37	0.02	(0.17–0.21)	p = 0.82
Model B	0.31	0.40	0.09	(0.09–0.26)	p = 0.34
**BAP (μg/l)**					
Model A	3.4	4.7	1.3	(-8.0–5.5)	p = 0.70
Model B	1.0	6.9	5.9	(-12.2–0.4)	p = 0.07

## Discussion

In the present analysis of the MINOXIS trial, the effects of treatment with low- versus high-flux dialysis membranes on circulating FGF-23 concentrations were studied. To the best of our knowledge, this is the first study investigating this issue in patients undergoing HD. The main finding was that over a period of 12 months, FGF-23 levels remained stable in the high-flux dialysis group, whereas an increase was observed in the low-flux dialysis group. The results were independent of potentially confounding variables including age, sex, C-reactive protein, albumin, calcium, phosphate, 25(OH)D and PTH levels.

FGF-23 acts through activation of the *klotho* receptor [11]. In the kidney, activation of the *klotho* receptor has phosphaturic effects *via* inhibition of proximal tubular phosphate reabsorption through sodium-dependent transporters [11, 12]. In HD patients, levels of FGF-23 are up to 1000 times higher compared with the normal population [7]. It is thought that the early increase in FGF-23 levels in CKD patients is an adaptive mechanism for preventing phosphate overload [4, 13]. Regarding the cardiovascular system, FGF-23 has been shown to induce left ventricular hypertrophy [14, 15], vascular calcification [16], arterial stiffness and endothelial dysfunction [17]. A recent study by Faul and colleagues demonstrated that intraventricular injection of FGF-23 directly induces pathological hypertrophy of the heart in rats [15]. These cardiovascular effects might explain why increased FGF-23 level has been found to independently predict CV mortality in CKD patients [18]. As a consequence of these detrimental effects of FGF23, there is pressing need to identify potential therapeutic options to lower concentrations of FGF-23 in patients with CKD.

The present results of the MINOXIS study demonstrate that high-flux HD was associated with stable FGF-23 concentrations over 12 months. Interestingly, enhanced removal of FGF-23 has been demonstrated in patients with on-line high-efficiency hemodiafiltration compared to both low-flux and high-flux dialysis [19, 20]. This finding suggests a more intense elimination by high-flux HD as a possible explanation for the differences in the FGF-23 levels observed in the MINOXIS study.

Similar to FGF-23, the secondary endpoint variable PTH has a predictive value with regard to mortality and CV events in HD patients [21, 22]. In the present study, paralleling the course of FGF-23, a significant increase of the PTH concentrations in the low-flux group but not in the high-flux group was observed. This observation is in line with a prospective study in 44 children on renal replacement therapy (RRT), which demonstrated more efficient PTH removal of high-flux compared to low-flux dialysis membranes [23]. PTH is a free circulating low-molecular weight protein (LMWP) with 9.500 Dalton [9] and, thus, easily permeable through high-flux membranes, but not through low-flux membranes. Nevertheless, the difference in the absolute change of the PTH levels between the two randomized groups was not significant.

The main limitation of the present study is its post hoc nature. The main strengths are the strict randomized design, the relatively large and well defined cohort, and the measurement of a large number of parameters of bone and mineral metabolism, permitting the statistical adjustment for a large number of potential confounders of the effects of dialysis membrane characteristics on FGF-23 levels.

In conclusion, over a period of 12 months, high-flux HD was associated with stable FGF-23 levels whereas the low-flux HD group showed an increase of FGF-23. However, FGF-23 levels remained excessively high in patients treated with high-flux dialysis. Thus, the clinical implications of different FGF 23 time-trends in patients on high flux versus low-flux dialysis remain to be explored in specifically designed clinical trials.

## Supporting Information

S1 Fig FGF-23 levels before and after 12 months of low-flux HD. (TIFF)

S2 Fig FGF-23 levels before and after 12 months of high-flux HD. (TIFF)
