# Supplementary figures and images for: Effect of High-Flux Dialysis on Circulating FGF-23 Levels in End-Stage Renal Disease Patients: Results from a Randomized Trial
Source: PLoS One. 2015 May 29;10(5):e0128079. doi: 10.1371/journal.pone.0128079 (PMC4449206; doi:10.1371/journal.pone.0128079)

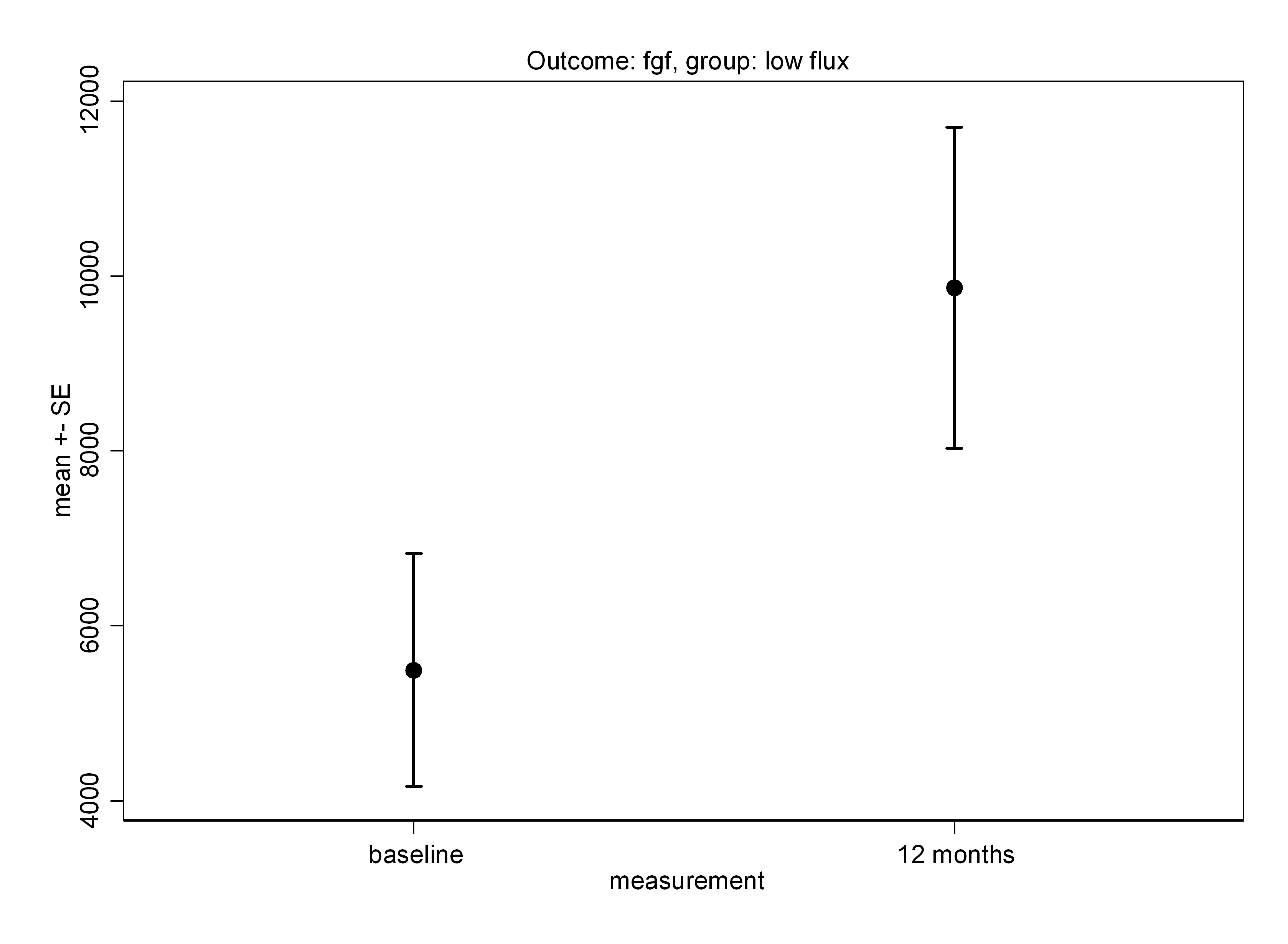

Supplement: S1 Fig — (TIFF) [file pone.0128079.s001.tiff]

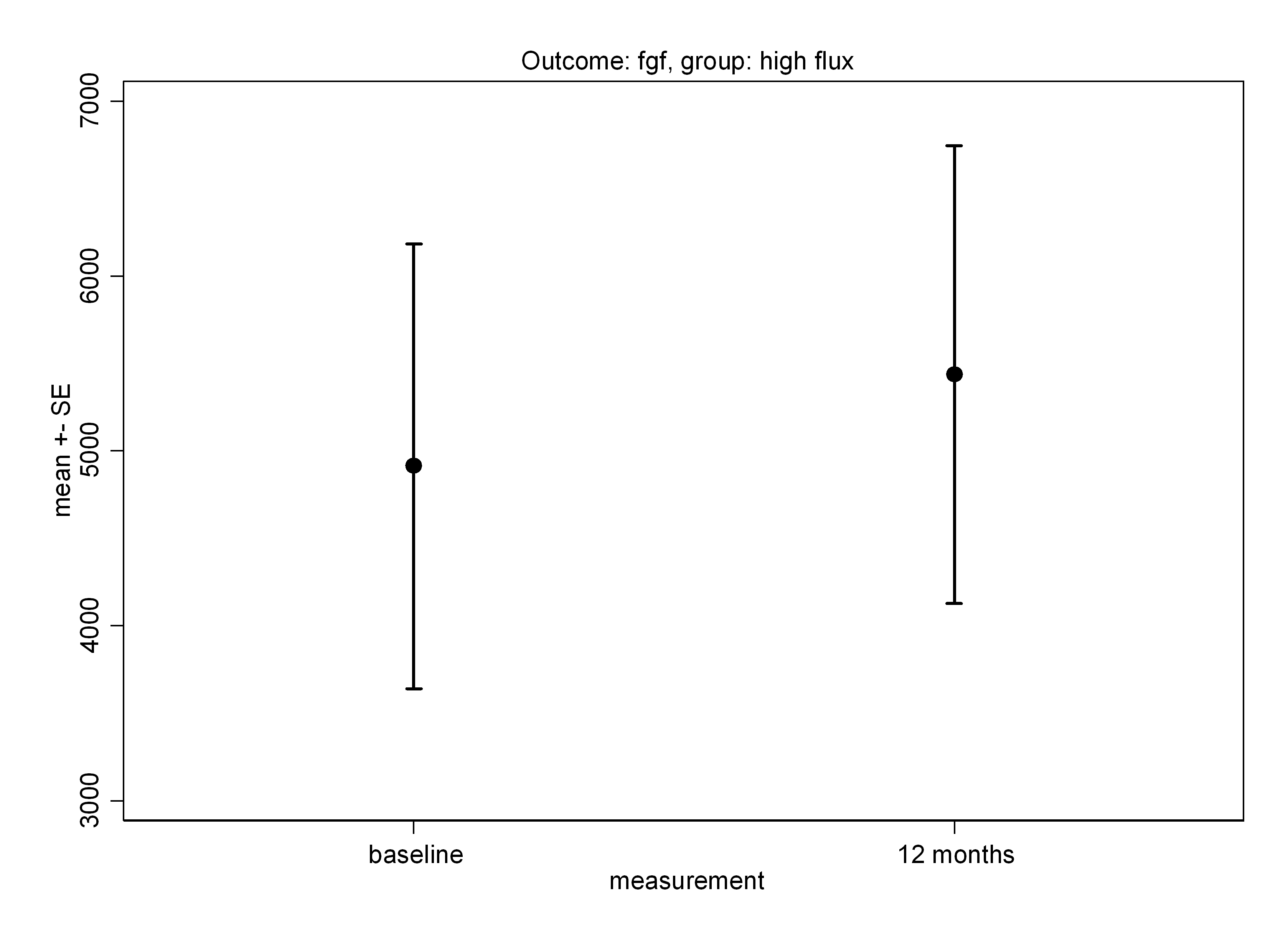

Supplement: S2 Fig — (TIFF) [file pone.0128079.s002.tiff]
